# Improving 3D-3D facial registration methods: potential role of three-dimensional models in personal identification of the living

**DOI:** 10.1007/s00414-021-02655-3

**Published:** 2021-07-09

**Authors:** Daniele Gibelli, Andrea Palamenghi, Pasquale Poppa, Chiarella Sforza, Cristina Cattaneo, Danilo De Angelis

**Affiliations:** 1 Dipartimento Di Scienze Biomediche Per La Salute, LAFAS, Laboratorio Di Anatomia Funzionale Dell’Apparato Stomatognatico, Università Degli Studi Di Milano, Via Mangiagalli 31, 20133 Milano, Italy; 2 Dipartimento Di Scienze Biomediche Per La Salute, LABANOF, Laboratorio Di Antropologia E Odontologia Forense, Università Degli Studi Di Milano, Via Mangiagalli 37, 20133 Milano, Italy

**Keywords:** Personal identification, Video surveillance system, Stereophotogrammetry, 3D-3D registration

## Abstract

Personal identification of the living from video surveillance systems usually involves 2D images. However, the potentiality of three-dimensional facial models in gaining personal identification through 3D-3D comparison still needs to be verified. This study aims at testing the reliability of a protocol for 3D-3D registration of facial models, potentially useful for personal identification. Fifty male subjects aged between 18 and 45 years were randomly chosen from a database of 3D facial models acquired through stereophotogrammetry. For each subject, two acquisitions were available; the 3D models of faces were then registered onto other models belonging to the same and different individuals according to the least point-to-point distance on the entire facial surface, for a total of 50 matches and 50 mismatches. RMS value (root mean square) of point-to-point distance between the two models was then calculated through the VAM® software. Intra- and inter-observer errors were assessed through calculation of relative technical error of measurement (rTEM). Possible statistically significant differences between matches and mismatches were assessed through Mann–Whitney test (*p* < 0.05). Both for intra- and inter-observer repeatability rTEM was between 2.2 and 5.2%. Average RMS point-to-point distance was 0.50 ± 0.28 mm in matches, 2.62 ± 0.56 mm in mismatches (*p* < 0.01). An RMS threshold of 1.50 mm could distinguish matches and mismatches in 100% of cases. This study provides an improvement to existing 3D-3D superimposition methods and confirms the great advantages which may derive to personal identification of the living from 3D facial analysis.

## Introduction

The analysis and comparison of faces are fundamental for the identification of the living from video surveillance systems: in this case, facial characteristics of a culprit recorded by cameras and a possible suspect are compared to verify the possible match [1]. At the state of the art, the gold standard for facial identification is represented by the 2D-3D superimposition, where a 3D model of the suspect is superimposed on the 2D images of the culprit from the video surveillance records [2, 3]. This procedure has been undoubtedly strongly developed thanks to the diffusion of 3D image acquisition systems, including sterephotogrammetry and laser scanners, which allow operators to extract three-dimensional models of the face of suspects [4, 5]. The obtained 3D model can then be moved and superimposed onto the images taken from the videosurveillace systems, so reducing the possible bias due to difference in orientation between the head position of the culprit and the suspect [6].

Although the 2D-3D superimposition procedure is the most used approach for a reliable comparison between facial characteristics of the culprit and the suspect, it is affected by some issues, being the most critical the quantification of differences between the two facial silhouettes and the consequent impossibility of expressing the judgement of identification in a numerical way. An attempt was performed by Yoshino et al. who analysed linear distances between facial landmarks identified on the 3D model of the suspect and on the 2D image of the culprit [7]. The analysis performed on 16 landmarks has some issues: the most critical one concerns bias in landmarks collocation which proved to vary among measurements taken by the same observers and among different operators according to the type of landmark [8]. Moreover, the method by Yoshino et al. showed overlapping distances between matches and mismatches [7], so preventing a definitive conclusion about match or mismatch.

The issue of quantification and the need for reliable methods able to distinguish matches from mismatches remain fundamental in the topic of identification from video surveillance systems. From this point of view, the progressive diffusion of 3D image acquisition systems may provide an innovative contribution, being able to provide a three-dimensional facial model, useful for further procedures of comparison [3, 9–13]. Therefore, techniques for 3D-3D superimposition need to be analysed in depth to verify the applicability of this novel technique of analysis to personal identification. From this point of view, a pilot study was published in 2017 by our research group, testing the potential of 3D-3D superimposition for personal identification: results were promising, although the procedure was based on landmark collocation and was tested only on 20 matches [14].

The present study aims at proposing a novel ameliorated protocol for 3D-3D registration, tested on 50 matches and 50 mismatches. Results will provide an important starting point for verifying if 3D-3D registration techniques brings about advantages in comparison to traditional 2D-3D methods, potentially useful for personal identification.

## Materials and methods

### Sample description

Fifty male adults, aged between 18 and 45 years (average age: 25.1 ± 7.0 years), were selected from a database of 3D facial models. The database includes 3D facial models collected by the Laboratory of Functional Anatomy of the Stomatognathic System (LAFAS) – Department of Biomedical Sciences for Health. Exclusion criteria were congenital or acquired pathologies potentially altering facial morphology, presence of beard, piercings or jewellery influencing the stereophotogrammetric acquisition, obesity. Moreover, the 50 selected subjects were not familiarly related.

For each individual, two 3D facial models were available in neutral position to exclude the possible influence of voluntary facial movement. All the subjects were acquired through stereophotogrammetric devices (VECTRA-3D**®** M3: Canfield Scientific, Inc., Fairfield, NJ) between 2014 and 2019. Technical characteristics of VECTRA-3D® M3 are as follows: sample density, 1.2 mm geometric resolution; capture volume, 400 × 300 × 250 mm; speed of acquisition, 3.5 ms [15].

Time span ranging between the two acquisitions was between 5–10 min and 50 months. In details, in 50% of cases time span was 5–10 min, in 26% of cases 1–12 months, and in 24% of cases 13–50 months. Subjects who had undergone maxillofacial, orthognathic or orthodontic surgery, as well as any other surgery potentially affecting facial morphology, were not included in the study. Two groups of 3D models were so created, the first including the earliest acquisition (group A) and the second one the latest one (group B). The study follows the guidelines provided by Helsinki Declaration and was approved by the University ethical committee (26.03.14; no. 92/14).

### 3D elaboration

**Fig. 1:**
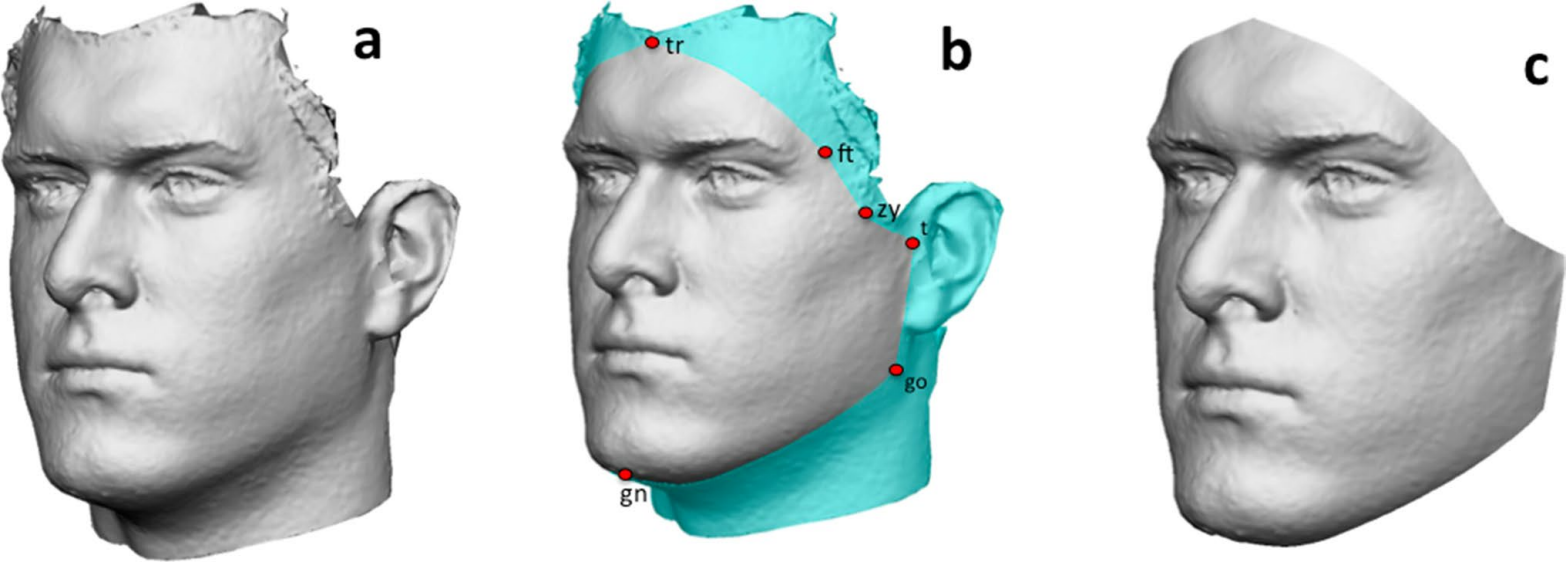
Phases of FAI selection: **a** acquired 3D model; **b** collocation of trichion (tr), right and left frontotemporale (ft), right and left zygion (zy), right and left tragion (tr), right and left gonion (go) and gnathion (gn); **c** resulting FAI (facial area of interest)

The chosen models were then elaborated through VAM® software (Canfield Scientific, Inc., Fairfield, NJ). From each 3D model, a FAI (facial area of interest) was selected, including the facial surface included between the trichion, right and left frontotemporale, right and left zygion, right and left tragion and gnathion landmarks (Table 1, Fig. 1). Then, FAI from 3D models belonging to group A and B were registered one on each other according to the following protocol:

**Table 1:** Abbreviation and definition of landmarks used for FAI selection and preliminary registration of 3D facial models

Landmark	Abbreviation	Definition
Trichion	tr	Midpoint of the hairline
Frontotemporale	ft	Deepest point of the frontotemporal fossa
Zygion	zy	Most lateral point of the zygomatic arch
Tragion	t	Superior point of the tragus incisure
Gonion	go	Most lateral point of the mandibular angle
Gnathion	gn	Most inferior point of the mandible in the midline
Exocanthion	ex	Lateral point of each palpebral fissure

-At first, the compared FAIs were oriented according to the least point-to-point distance according to the right and left exocanthion and gnathion landmarks;

-A registration was performed according to the least point-to-point distance on the entire surface; this procedure was automatically performed by the 3D analysis software; both the preliminary and final registrations were performed using the model from group A as the reference one (i.e. the 3D model from group B was moved and superimposed on the 3D model from group A).

**Fig. 2:**
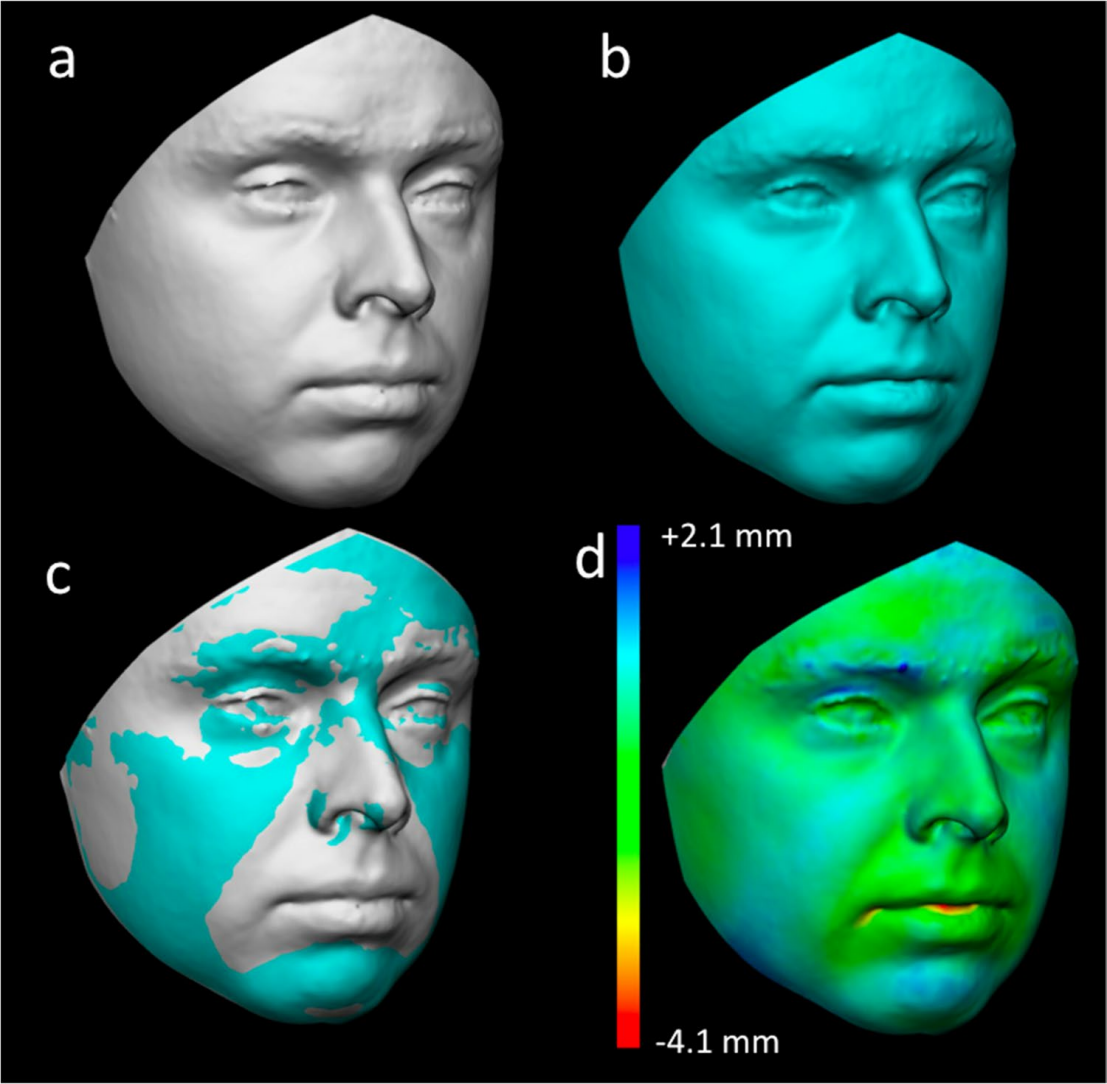
3D-3D registration of 3D models belonging to the same individual (match): **a** 3D model from group A; **b** 3D model from group B; **c** registration of the two 3D models according to the least point-to-point distance between the entire surfaces; **d** chromatic visualisation of linear differences between the two models: in green, coincident points between the two models: in light and dark blue, recessing areas of model from group B according to model from group A: in red and yellow, protruding areas of model from group B according to model from group A

**Fig. 3:**
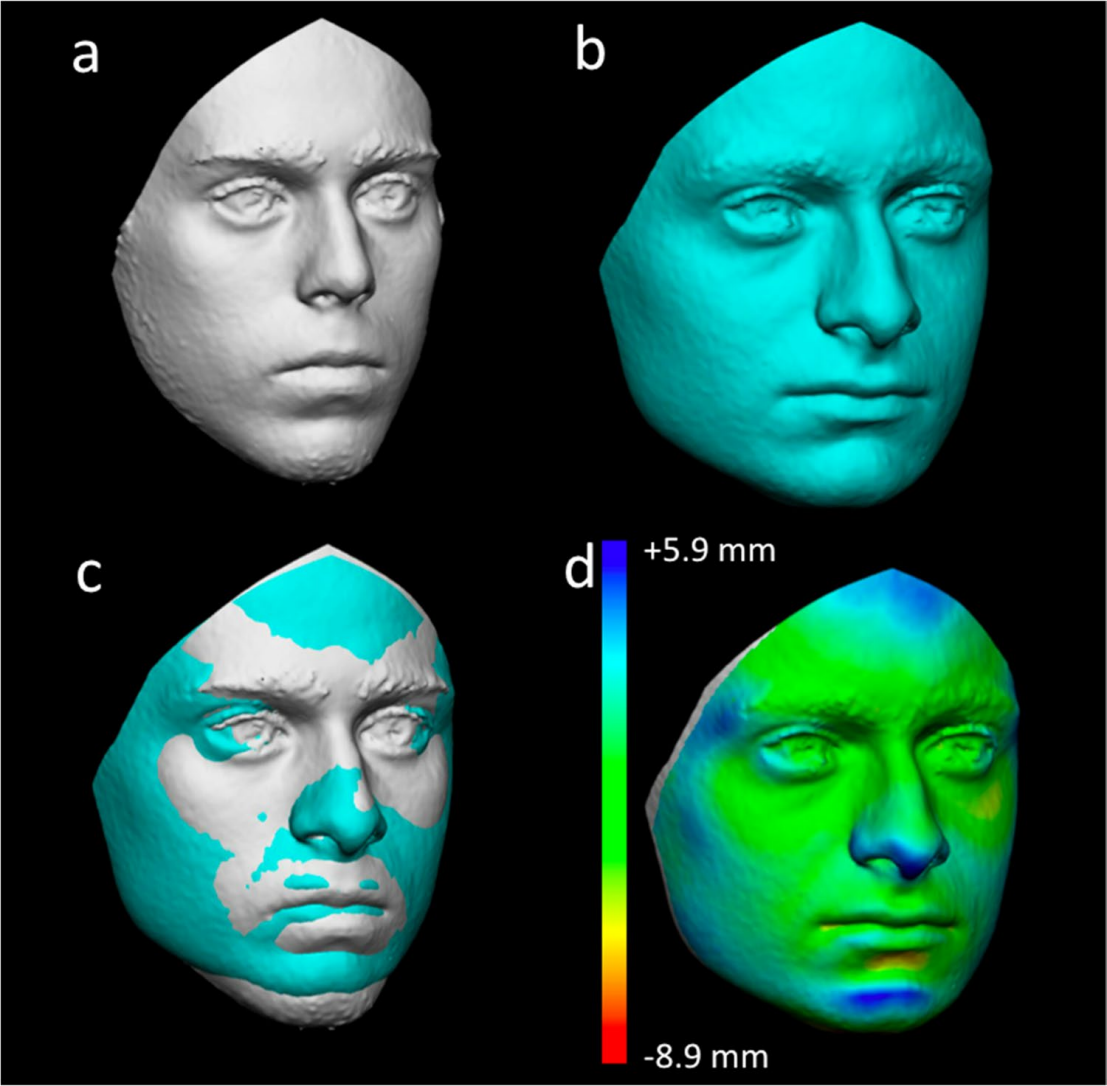
3D-3D registration of 3D models belonging to different individuals (mismatch): **a** 3D model from group A; **b** 3D model from group B; **c** registration of the two 3D models according to the least point-to-point distance between the entire surfaces; **d** chromatic visualisation of linear differences between the two models: in green, coincident points between the two models: in light and dark blue, recessing areas of model from group B according to model from group A: in red and yellow, protruding areas of model from group B according to model from group A

-RMS (root mean square) point-to-point distance between the two FAIs was then automatically calculated by the 3D analysis software. The calculation of RMS point-to-point was performed taking the FAI of group A as the reference one. Together with the calculation of RMS values, the 3D analysis software provides a chromatic visualisation of distances between the points of the two registered models (in green, coincident points between the two models: in light and dark blue, recessing areas of model from group B according to model from group A: in red and yellow, protruding areas of model from group B according to model from group A). Each 3D model was registered onto the 3D model belonging to the same individual to create a group of 50 matches. In addition, among the possible combinations, other 50 registrations were randomly performed between 3D models belonging to different individuals to create the mismatches group (Figs. 2 and 3).

All the elaboration steps of the 3D facial models from the FAI selection to registration and calculation of RMS point-to-point distance were performed by two authors with plurennial experience in 3D analysis.

### Statistical analyses

To verify possible influence of time span on facial differences found in the same individual, Pearson’s correlation index was calculated between the time passing between the two scans and the RMS values in the matches group (*p* < 0.05).

The entire procedure of 3D-3D registration from the selection of FAI to the calculation of RMS point-to-point distance was repeated for ten matches and ten mismatches by the same operator after two weeks and by a different operator, to test intra- and inter-observer error, respectively. To assess the possible influence of FAI selection on results, intra- and inter-observer error was calculated also for the areas of selected FAI in 25 3D models. For both the parameters, error was expressed as relative technical error of measurement (rTEM) [16].

Possible statistically significant differences between matches and mismatches were assessed through Mann–Whitney test (*p* < 0.05).

## Results

The proposed protocol was found to be highly repeatable, with an intra- and inter-observer error of 3.5% and 5.2% in matches, and of 2.2% and 4.6% in mismatches, respectively (Table 2). In all the cases, repeatability could be classified as ‘good’ and ‘very good’ according to Camison et al. [4]. Similar results were found for areas of FAIs, being the intra- and inter-observer error 2.9% and 3.6%, respectively; repeatability of both the parameters could be classified as as ‘very good’ [4].

**Table 2:** Intra- and inter-observer error for matches and mismatches, expressed as TEM (absolute technical error of measurement) and rTEM (relative technical error of measurement)

	Intra-observer error		Inter-observer error	
	TEM (mm)	rTEM (%)	TEM (mm)	rTEM (%)
Matches	0.01	3.5	0.02	5.2
Mismatches	0.06	2.2	0.12	4.6

No significant correlation was found between time span and RMS values in matches group (correlation index, 0.27; *p*, 0.061).

RMS values in matches ranged between 0.17 and 1.38 mm, with a mean of 0.50 ± 0.28 mm. In mismatches, RMS values ranged between 1.74 and 4.28 mm, with a mean of 2.62 ± 0.56 mm. No overlaps were found between the RMS values obtained by matches and mismatches, the latters always being larger than the formers. Differences between matches and mismatches were statistically different (*p* < 0.01).

**Fig. 4:**
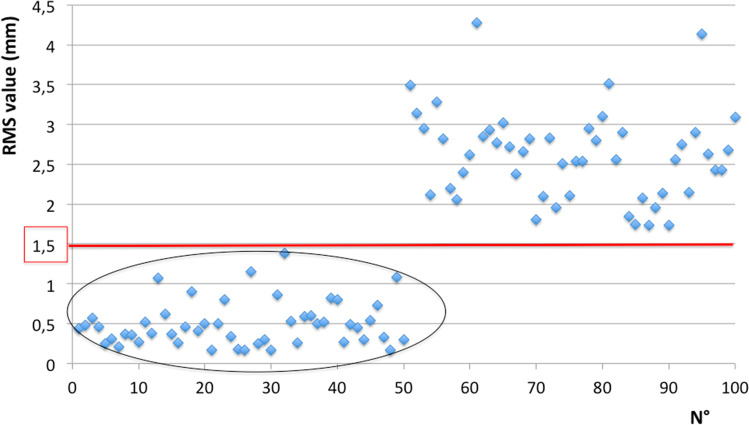
distribution of RMS values: within the black circle the group of matches

Analysing the distribution of data, an arbitrary threshold of 1.50 mm could distinguish matches and mismatches in 100% of cases (Fig. 4).

## Discussion

Personal identification from video surveillance systems has always brought about specific issues in forensic practice, being the most critical the quantification of differences between facial silhouettes. 2D-3D superimposition represents the most reliable procedure for a comparison between facial features of possible suspects and the culprit recorded in surveillance system [3, 7]. However, the few quantitative methods to assess possible matches are based on facial landmarks (whose collocation is affected by intra- and inter-operator variability) and highlight a partial overlapping between matches and mismatches [7].

The topic of the present article was to verify if a 3D-3D comparison provides an amelioration of superimposition procedures, with a higher chance of distinguishing between matches and mismatches in comparison with commonly used methods where 3D models are superimposed on bi-dimensional images extracted from videosurveillace systems: the advantages of the described protocol are that it is almost completely independent from collocation of facial landmarks (but for the selection of FAI and the preliminary registration) and it shows high intra- and inter-operator repeatability which concerns not only automatic procedures of registration and calculation of RMS values but also semi-automatic step of FAI selection. In fact, both in matches and mismatches the intra- and inter-observer repeatability of RMS calculation was between 2.2 and 5.2%, and slightly higher in the former than in the latter cases: an explanation may be the lower RMS values reported in matches than in mismatches; in fact, literature reports that the measurements with the smallest magnitude are usually less repeatable [17]. This phenomenon is observed also in other repeatability analyses involving small measurements [18]. Also, the selection of FAI proved to be highly repeatable, with both an intra- and inter-observer error classified as ‘very good’ according to the literature [4].

Moreover, the proposed method represents a clear improvement in comparison with the pilot procedure exposed by the same research group in 2017: in fact, in the previous procedure, registration was entirely performed according to the least distance between the two models according to nine landmarks, so resulting in a less adherent registration with consequent higher RMS values [14]. In the present study, the registration was performed, after a preliminary orientation through three landmarks, according to the least point-to-point distance on the entire surface of compared models. This amelioration increased the repeatability of the procedure, as registration is automatically performed by the 3D analysis software. Another important improvement of the present protocol concerns the clear distinction between matches and mismatches according to RMS values, whereas the previous method showed an overlap between matches and mismatches [14].

The present study clearly shows that the comparison of two 3D facial models may represent a crucial improvement of personal identification of the living, comparing two 3D models of faces from both the suspect and the culprit. Obviously, the main limit to the extensive use of this procedure is the impossibility of acquiring a 3D model of faces from video surveillance systems: in fact the resolution of existing cameras is inadequate to provide a 3D model with a sufficient accuracy for the following 3D-3D registration procedures. Moreover, we have to consider that the choice of installing specific video-surveillance systems does not depend only upon the quality of given images, but also on economical (influence of the cost of different devices) or logistic (for example, choosing to increase the number of checked areas through several low-quality cameras, instead of focusing on a specific zone with high-quality devices) factors. From this point of view, although technical innovations come at high speed, especially in the field of 3D image acquisition and analysis, the fast adoption of modern 3D video surveillance systems is yet to come and probably will not be available in the next future. Anyway, the results of the present study show that such improvement is auspicable, as it may provide a crucial development of modern methods for personal identification of the living.

The present protocol is affected by some limits: first of all, results may depend upon the type of 3D image acquisition systems and the 3D analysis software. Although the reliability of the used stereophotogrammetric system (VECTRA-3D**®**: Canfield Scientific, Inc., Fairfield, NJ) has been widely verified by literature [19, 20], several technologies are available for 3D image acquisition (laser scanner, structured light techniques, etc.) which may lead 3D models with different technical characteristics [15, 21, 22]. In fact, literature suggests caution in registering 3D models acquired through different types of device (i.e. from stereophotogrammetry and laser scanner), as the RMS values increase [5].

Moreover, also, the reproducibility of results among different 3D analysis softwares needs to be verified. The future studies will focus on reproducibility of results deriving from 3D-3D registration procedures among different 3D image acquisition systems and softwares.

Finally, another important point to explore concerns the possible influence of voluntary facial mimicry on RMS values obtained through 3D-3D registration: in fact, although involuntary facial movements cannot be avoided, in forensic practice, sometimes facial images are compared representing different facial expressions. Again, future studies should verify how facial mimicry may affect results deriving from 3D-3D registration of facial models belonging to the same individual.

In conclusion, the present study describes an innovative method for personal identification from 3D-3D registration of 3D facial models. Results show that the 3D morphology of faces may be sufficient to identify living subjects. We trust that the technological evolution will soon enable operators to apply this technique, so improving the reliability of existing methods for personal identification of the living.

## References

[1] Gibelli D, Obertovà Z, Ritz-Timme S, Gabriel P, Arent T, Ratnayake M, De Angelis D, Cattaneo C (2016). The identification of living persons on images: a literature review. Leg Med.

[2] De Angelis D, Sala R, Cantatore A, Poppa P, Dufour M, Grandi M, Cattaneo C (2007). New method for height estimation of subjects represented in photograms taken from video surveillance systems. Int J Legal Med.

[3] Atsichi M, Tsuji A, Usumoto Y, Yoshino M, Ikeda N (2013). Assessment of some problematic factors in facial image identification using a 2D/3D superimposition technique. Leg Med.

[4] Camison L, Bykowski M, Lee W, Carlson JC, Roosenboom J, Goldstein JA, Losee JE, Weinberg SM (2018). Validation of the Vectra H1 portable three-dimensional photogrammetry system for facial imaging. Int J Oral Maxillofac Surg.

[5] Gibelli D, Pucciarelli V, Poppa P, Cummaudo M, Dolci C, Cattaneo C, Sforza C (2018). Three-dimensional facial anatomy evaluation: reliability of laser scanner consecutive scans procedure in comparison with stereophotogrammetry. J Craniomaxillofac Surg.

[6] Yoshino M, Matsuda H, Kubota S, Imaizumi K, Miyasaka S (2000). Computer-assisted facial image identification system using a 3-D physiognomic range finder. Forensic Sci Int.

[7] Yoshino M, Matsuda H, Kubota S, Imaizumi K, Kiyasaka S (2000). Assessment of computer-assisted comparison between 3D and 2D facial images. Jpn J Sci Tech Iden.

[8] Cummaudo M, Guerzoni M, Marasciuolo L, Gibelli D, Cigada A, Obertovà Z, Ratnayake M, Poppa P, Gabriel P, Ritz-Timme S, Cattaneo C (2013). Pitfalls at the root of facial assessment on photographs: a quantitative study of accuracy in positioning facial landmarks. Int J Legal Med.

[9] Nightingale RC, Ross MT, Allenby MC, Woodruff MA, Powell SK (2020). A method for economical smartphone-based clinical 3D facial scanning. J Prosthodont.

[10] Farook TH, Rashid F, Jamayet NB, Abdullah JY, Dudley J, Alam MK (2021) A virtual analysis of the precision and accuracy of 3-dimensional ear casts generated from smartphone camera images. J Prosthet Dent [Epub ahead of print] 10.1016/j.prosdent.2020.12.041 33642077

[11] Leipner A, Obertovà Z, Wermuth M, Thali M, Ottiker T, Sieberth T (2019). 3D mug shot – 3D head models from photogrammetry for forensic identification. Forensic Sci Int.

[12] Bonnechère B, Jansen B, Salvia P, Bouzahouene H, Sholukha V, Cornelis J, Rooze M, Van Sint JS (2014). Determination of the precision and accuracy of morphological measurements using the Kinect sensor: comaprison with standard stereophotogrammetry. Ergonomics.

[13] Gibelli D, Pucciarelli V, Cappella A, Dolci C, Sforza C (2018). Are portable stereophotogrammetric devices reliable in facial imaging? A validation study of VECTRA H1 device. J Oral Maxillofac Surg.

[14] Gibelli D, De Angelis D, Poppa P, Sforza C, Cattaneo C (2017). A view to the future: a novel approach for 3D–3D superimposition and quantification of differences for identfication from next-generation video surveillance systems. J Forensci Sci.

[15] Tzou CHJ, Artner NM, Pona I, Hold A, Placheta E, Kropatsch WG, Frey M (2014). Comparisons of three-dimensional surface imaging systems. J Plast Reconstr Aesthet Surg.

[16] Adao Perini T, Lameira de Oliveira G, dos Santos OJ, Palha de Oliveira F (2005). Technical error of measurement in anthropometry. Rev Bras Med Esporte.

[17] Jamison PL, Ward RE (1993). Brief communication: measurement size, precision, and reliability in craniofacial anthropometry: bigger is better. Am J Phys Anthropol.

[18] Gibelli D, Cappella A, Bertozzi F, Sala D, Sitta S, Tasso DR, Tomasi F, Dolci C, Sforza C (2021) Three-dimensional facial anthropometric analysis with and without landmark labelling: is there a real difference? J Craniofac Surg [Epub ahead of print] 10.1097/SCS.0000000000007687 PMC10292572 33867510

[19] de Menezes M, Rosati R, Ferrario VF, Sforza C (2010). Accuracy and reproducibility of a 3-dimensional stereophotogrammetric imaging system. J Oral Maxillofac Surg.

[20] Rosati R, De Menezes M, Rossetti A, Sforza C, Ferrario VF (2010). Digital dental cast placement in 3-dimensional, full-face reconstruction: a technical evaluation. Am J Orthod Dentofacial Orthop.

[21] Gibelli D, Dolci C, Cappella A, Sforza C (2020). Reliability of optical devices for three-dimensional facial anatomy description: a systematic review and meta-analysis. Int J Oral Maxillofac Surg.

[22] Gibelli D, Pucciarelli V, Caplova Z, Cappella A, Dolci C, Cattaneo C, Sforza C (2018). Validation of a low-cost laser scanner device for the assessment of three-dimensional facial anatomy in living subjects. J Craniomaxillofac Surg.

